# Clinical Knowledge and Trends in Physicians' Prescribing of Opioids for New Onset Back Pain, 2009-2017

**DOI:** 10.1001/jamanetworkopen.2021.15328

**Published:** 2021-07-01

**Authors:** Bradley M. Gray, Jonathan L. Vandergrift, Weifeng Weng, Rebecca S. Lipner, Michael L. Barnett

**Affiliations:** 1 American Board of Internal Medicine, Philadelphia, Pennsylvania; 2 Harvard School of Public Health, Boston, Massachusetts

## Abstract

**Importance:**

Opioid musculoskeletal pain overprescribing was widespread in the mid-2000s. The degree to which prescribing changed as awareness of the danger grew among physicians with different levels of clinical knowledge remains unstudied.

**Objective:**

To compare the association of clinical knowledge with opioid prescribing from 2009 to 2011 when prescribing peaked nationally with 2015 to 2017 when guidelines shifted away from opioid prescribing.

**Design, Setting, and Participants:**

This cross-sectional study included 10 246 midcareer general internal medicine physicians in the United States who saw patients who were Medicare beneficiaries with Part D enrollment from 2009 to 2017.

**Main Outcomes and Measures:**

Any opioid prescription and high dosage or long duration (HDLD) (>7 days or >50 daily morphine milligram equivalents) opioid prescriptions filled within 7 days of applicable visits for new low back pain concerns. Associations between opioid prescribing for new low back pain concerns during outpatient visits and clinical knowledge measured by prior year American Board of Internal Medicine (ABIM) Maintenance of Certification examination performance were estimated using serial cross-sectional logit regressions. Regression covariates included yearly examination quartile (ie, knowledge quartile) interacted with 3-year group dummies (ie, early: 2009-2011; middle: 2012-2014; late: 2015-2017), state and year dummies, physician, practice, patient characteristics, and state opioid regulations.

**Results:**

Of the 55 387 low back pain visits included in this study, 37 185 (67.1%) were visits with female patients, 41 978 (75.8%) were with White patients, and the mean (SE) age of patients was 76.2 (<0.01) years. The rate of opioid prescribing was 21.6% (11 978) for any opioid prescription and 17.6% (9759) for HDLD prescriptions. From 2009 to 2011, visits with physicians in the highest and lowest knowledge quartiles had similar adjusted opioid prescribing rates with a 0.5 (95% CI, –1.9 to 3.0) percentage point difference. By 2015 to 2017, visits with physicians in the highest knowledge quartile prescribed opioids less frequently that physicians in the lowest knowledge quartile (4.6 percentage point difference; 95% CI, –7.5 to –1.8 percentage points). Visits in which HDLD opioids were prescribed showed no difference in the early period but showed a difference in the late period when comparing physicians in the highest and lowest knowledge quartiles (early period: difference –0.1; 95% CI, –2.4 to 2.2 percentage points; late period difference: 4.8; 95% CI, –7.4 to –2.1 percentage points).

**Conclusions and Relevance:**

In this cross-sectional study, when the standard of care shifted away from routine opioid prescribing, physicians who performed well on an ABIM examination were less likely to prescribe opioids for back pain than physicians who performed less well on the examination.

## Key Points

**Question:**

Is clinical knowledge associated with opioid prescribing and has this association changed over time?

**Findings:**

In this cross-sectional study from 2009 to 2017 of 10 246 physicians, opioids were prescribed during 21.6% new onset low back pain office visits. From 2015 to 2017, opioid prescription rates were 4.6 percentage points lower in visits with physicians in the highest vs lowest quartile of performance on American Board Internal Medicine’s Maintenance of Certification examination despite there being no difference in the earlier 2009 to 2011 or 2012 to 2014 periods.

**Meaning:**

These findings suggest that physicians with higher clinical knowledge scores had reduced opioid prescribing in 2015 to 2017, when guidelines were rapidly changed toward reduced opioid prescribing.

## Teaser

This cross-sectional study compares the association of clinical knowledge with opioid prescribing from 2009 to 2011 when prescribing peaked nationally with 2015 to 2017 when guidelines shifted away from opioid prescribing.

## Introduction

Opioid overprescribing in the early 2000s is considered to be a major contributor to the genesis of the modern opioid use disorder crisis.^1,2,3,4^ Experts believe that opioid overprescribing was fueled in part by pharmaceutical manufacturer–supported advocacy that pain was widely undertreated and that opioids, especially oxycodone, were marketed to primary care physicians as a safe and effective means to reduce unnecessary pain.^3,5,6,7,8^ This advocacy was accompanied by broad guideline changes during the late 1990s and early 2000s that promoted the use of opioids for pain management. A near doubling of outpatient opioid prescriptions followed between 2000 and 2011, with opioids being prescribed most commonly to patients who were age 65 years or older.^9,10^ Despite this growth in prescribing, the actual evidence that opioid therapy was more effective for pain control than nonopioid alternatives was extremely limited.^11,12,13^

After 2011, more evidence pointed to the routine prescribing of opioids as likely contributing to a dramatic increase in opioid-related deaths.^14^ The prevailing culture toward opioid prescribing began to shift to the point where, by 2015, leading medical societies were changing their guidelines and guidance to recommend against routine prescribing of opioids for pain in favor of safer alternatives.^14,15,16,17,18,19^ For example, in 2015, the American Academy of Orthopedic Surgeons released an advisory outlining new guidelines for opioid prescriptions for muscular skeletal conditions warning of the dangers of opioid prescriptions.^15^ In 2016, the US Centers for Disease Control and Prevention published widely circulated opioid prescribing guidelines for chronic pain, and the US Food and Drug Administration had begun requiring a new boxed warning for opioid medications detailing their serious risks of addiction and overdose.^19^ In 2017, the American College of Physicians changed its opioid prescribing guidelines that had been in place since 2007 and argued against opioid use for lower back pain (LBP).^17^ Despite this substantial shift in guidelines and growing evidence raising concern about routine opioid use, opioid prescribing remains highly variable among physicians.^20,21^

We hypothesized that a contributor to this variation is that physicians with higher clinical knowledge stay up-to-date with medical research and are more responsive to these changes in guidelines and evidence. To address this hypothesis, we measured the association between a general internal medicine physician’s performance on the American Board of Internal Medicine’s (ABIM) internal medicine board examination and opioid prescribing for their Medicare patients’ new low back pain concern between 2009 and 2017, comparing associations during the early study period (ie, 2009-2011), when the dangers of opioid prescribing were just emerging, to the later period (ie, 2015-2017), when standards of care had shifted.

## Methods

### Study Sample

The Advarra institutional review board approved our study protocol and informed consent was waived because the study was viewed as exempt. This study follows the Strengthening the Reporting of Observational Studies in Epidemiology (STROBE) reporting guideline.

We used the ABIM board certification database to identify all general internal medicine physicians (ie, nonsubspecializing internal medicine physicians) who were approximately 10 years past their initial certification and had taken a Maintenance of Certification (MOC) internal medicine examination from 2008 to 2016 (N = 26 187) (Figure). Using National Provider Identifier (NPI) codes acquired from the Federation of State Medical Boards (25 853 [98.7%]) and Medicare Carrier claims files, we linked these physicians to eligible Medicare fee-for-service outpatient LBP visits during the year after their MOC examination; this included 12 056 physicians and 202 991 LBP visits from 2009 to 2018 (eTable 1 in the Supplement).^22^ We chose this study period because when the study was conducted Medicare opioid prescription data was not available before 2009 or after 2017. For physicians with multiple examination attempts, we used their last examination to increase the accuracy of our knowledge measure. This is because studying for a subsequent attempt may increase knowledge in a way not reflected in the examination score. Using Medicare’s beneficiary summary file, we limited the sample to beneficiaries in the US with Part D plan drug coverage, during the month of the visit and prior 3 months to ensure complete claims and prescription data were available from 11 174 physicians and 122 179 visits.

**Figure.**
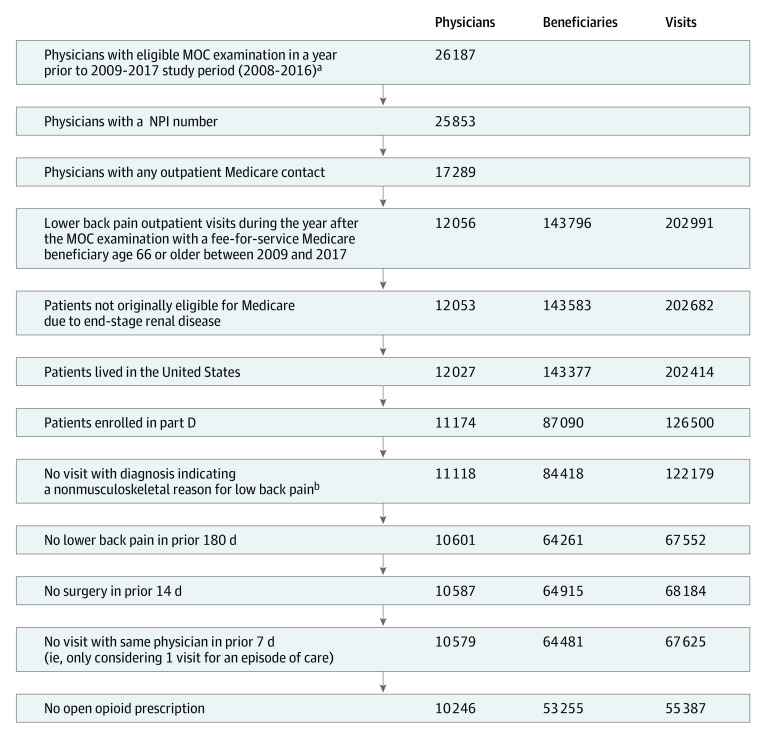
Sample Selection Abbreviations: MOC, maintenance of certification; NPI, National Provider Identification. ^a^Last examination attempt (ie, no examination in the following year). ^b^Kidney stones, gallbladder stones (no cholecystitis), urinary tract infection, cancer, osteoporosis, Cauda equine syndrome, osteomyelitis, or major osseous defect.

We then limited these visits to those with an eligible new low back pain concern, which were referred to as LBP visits, and defined by the recorded visit diagnosis for LBP without another such diagnosis within 180 days prior. Furthermore, visits that included a diagnosis that could be confused with nonmusculoskeletal LBP^22^ were excluded. This excluded 10 061 physicians and 67 552 visits ( eTable 1 in the Supplement). We also excluded visits with surgical procedures within the prior 14 days (because opioid prescriptions might have been appropriate) and visits where the patient had received a prior opioid prescription that was still open at the date of the visit. After these exclusions, we were left with 55 387 visits with 10 246 physicians in our final sample.

### Opioid Prescribing Outcomes

Using Medicare Part D files, we identified all opioid prescriptions filled within 7 days after the index LBP visit written by the physician who billed for that visit. In addition to any opioid prescription, we also classified prescriptions as either a high-dosage or long-duration (HDLD) prescription if it exceeded 50 daily morphine milligram equivalents (MME) or 7 days supplied or a low-dosage or short-duration (LDSD) prescription if it was a less than or equal to 50 daily MME and less than or equal to a 7-day supply.^23^ Opioid prescriptions and potency were identified using a Centers for Disease Control and Prevention (CDC) formulary.^24^

### Knowledge Measure

Our measure of physician knowledge was equated scores from the internal medicine MOC examination. Equating is a process to make scores comparable across adjacent examination administrations (eTable 2 and eTable 3 in the Supplement). Starting in the second half of 2008, examination scores were equated by ABIM using item response theory. These equated scores were not available for the first examination administration in 2008 and were estimated to reflect equating.^25^

ABIM designed examination questions to replicate decision-making during real-world clinical scenarios faced by general internal medicine physicians, primarily in the outpatient setting, rather than questions that required rote memorization.^26^ Scenarios used as a basis for questions reflected the wide variety of conditions treated by outpatient general internal medicine physicians.^27,28^ While the focus of the examination is not specifically pain management, appropriate pain management is an important component of clinical knowledge that the examination is designed to cover, and back pain is a topic explicitly included in the examination blueprint (ie, representing as much as 2% of the examination).^27^

Equated examination scores were categorized yearly by quartile using equated examination breaks based on ranking among physicians taking the examination for the first-time equated scores (eTable 3 in the Supplement). We used these cutoffs because the scores among first-time test takers are relatively consistent across time allowing for better comparison of physician ability over time.

### Statistical Analysis

We used visit-level logit regression to estimate associations between our opioid prescribing outcomes and knowledge quartile across time. Key regression covariates included indicators for knowledge quartile and their interactions with early (2009-2011), middle (2012-2014), and late (2015-2017) period dummies. Other covariates adjusted for visit year, state fixed effect, and time-varying state laws (prescription registration mandates and/or dosage duration limitations). Physician and practice characteristics from an MOC survey and administrative data were practice size (eg, solo, practices larger than 50 physicians), practice type (eg, academic medical facility, group practice, community health center, nursing home), training (ie, a physician’s US medical school location interacted with US birth country), and physician gender. Training characteristics, although they may be associated with physician knowledge, may also be related to unobserved patient characteristics (eg, some patients might prefer US-trained or US-born physicians). Patient covariates, from the Medicare Beneficiary Summary files, included sociodemographic characteristics, such as age and age squared, gender, race/ethnicity (ie, White not Hispanic, Black not Hispanic, Hispanic, Asian, and Other). Race and ethnicity were included in this study because it has been identified as an independent risk factor for opioid prescriptions.^29^ The covariates also included Medicaid eligibility during the study year and 1-year lagged measures of patient health risk (ie, 30 comorbid chronic conditions, including history of anxiety, depression, substance use disorder, and opioid use disorder; and the Elixhauser comorbidity index).^30^ Visit-level covariates controlled for health care use (ie, surgery, emergency department visits, and hospitalizations) in the prior 14, 30, 90, and 180 days and geographic characteristics (ie, US state, rural county, and ZIP-code level median household income). Medicare measures of race/ethnicity from the Beneficiary Summary file were primarily based on Social Security administrative data through self-reports.^31^

Applying the results of these regressions, we estimated adjusted knowledge quartile–associated differences within and across the early, middle, and late periods using the cross partial derivative method described by Karaca-Mandic et al.^32^ Regression adjusted means by quartile within year groups were constructed by assuming a given quartile and year group interaction, holding all other covariates constant, and estimating prescription rates over the visit sample. Differences across quartiles within and across year groups were then computed by differencing these estimates. This approach considers the nonlinearity of the logit models when interpreting interaction terms and calculating standard errors. Standard errors were also adjusted for visits being clustered within physicians.^33,34,35^

To frame our results, we simulated prescribing rates based on regression coefficient estimates and actual patient and physician characteristics and assuming all study sample physicians were in the highest knowledge quartile during the 3 years that comprise the late period. We then scaled up these estimates to reflect all clinically active outpatient general internal medicine physicians (eAppendix 2 in the Supplement). We performed the following sensitivity analyses: (1) reassigning visits occurring before the CDC opioid guideline changes on April 16, 2016, from the last to the middle period; (2) examining prescribing of potential alternative medications not available over the counter and appropriate for older age populations^36,37,38^; (3) excluding visits to physicians whose equated examination scores were estimated because they took their examination during the first part of 2008; (4) excluding physician training measures from the regression because they might be associated with clinical knowledge (eAppendix 3 in the Supplement). All analyses were performed using Stata software version 16 (StataCorp). Hypothesis tests were 2-tailed with *P* < .05 considered statistically significant.

## Results

### Sample

Of the 55 387 LBP visits that met our study criteria, 6697 visits (12.1%) were with patients from rural zip codes, 41 978 visits (75.8%) were with White patients, 37 185 visits (67.1%) were with female patients, and the mean (SD) patient age was 76.2 (<0.01) years. Of the included visits, 15 035 (27.1%) were with physicians in solo practices and 2806 (5.1%) in academic medical centers (Table 1). Weighted by number of LBP visits, physicians in the lowest knowledge quartile answered substantially fewer questions correctly than physicians in the highest quartile (mean [SE] MOC examination percent correct, 63.5% [0.3] vs 84.5% [0.1]). Physicians in the lowest knowledge quartile were more likely to be international medical school graduates and be in solo practice than physicians in the highest knowledge quartile (international medical school graduates: 8974 [64.0%] vs 3951 [36.9%]; solo practice: 5412 [38.6%] vs 1592 [14.8%]). Physicians in the lowest knowledge quartile were less likely to be in large practices with more than 25 physicians or academic medical centers than physicians in the highest quartile (large practice with >25 physicians: 1269 [9.0%] vs 2131 [19.9%]; or academic medical centers: 202 [1.4%] vs 1025 [9.6%]). Although sometimes statistically significant, there were relatively small differences in most visit-level characteristics of patients seen by physicians in different knowledge quartiles. For example, the number of physicians in the lowest knowledge quartile of visits with patients residing in rural areas compared with those in the highest knowledge quartile was 1430 (10.2%) vs 1344 (12.5%). An exception to this was the share of visits with White patients, for which physicians in the highest quartile had 8677 visits (80.9%) vs 9832 visits (70.1%) for physicians in the lowest knowledge quartile.

**Table 1.** Comparisons of Physician and Patient Characteristics Across Visits With Physicians by Knowledge Quartiles

Characteristic	Visits, No. (%)					*P* value
	Total	Knowledge quartiles^a^				
		1	2	3	4	
Physician characteristic						
Female	20 078 (36.3)	4442 (31.7)	6697 (37.2)	5116 (40.5)	3823 (35.7)	.007
International medical graduate,	29 017 (52.4)	8974 (64.0)	10 102 (56.1)	5990 (47.4)	3951 (36.9)	<.001
Practice size						
Solo practice	15 035 (27.1)	5412 (38.6)	5397 (30.0)	2634 (20.8)	1592 (14.8)	<.001
>25 physicians in the practice	7261 (13.1)	1269 (9.0)	2236 (12.4)	1625 (12.9)	2131 (19.9)	<.001
>50 physicians in the practice	4790 (8.6)	876 (6.2)	1410 (7.8)	1056 (8.4)	1448 (13.5)	<.001
Practice type						
Group practice	49 751 (89.8)	12 940 (92.2)	16 191 (90.0)	11 277 (89.2)	9343 (87.1)	.002
Community health center	349 (0.6)	97 (0.7)	129 (0.7)	82 (0.6)	41 (0.4)	.48
Academic practice	2806 (5.1)	202 (1.4)	862 (4.8)	717 (5.7)	1025 (9.6)	<.001
Visits, mean (SD), No.	142.2 (3.0)	158.5 (6.8)	142.8 (4.1)	137.6 (6.9)	125.5 (5.5)	.002
MOC examination percent correct, mean (SE)	73.2 (0.2)	63.5 (0.3)	71.0 (0.1)	77.4 (0.1)	84.5 (0.1)	<.001
Patient characteristics						
Age, mean (SE)	76.2 (0.0)	76.2 (0.1)	76.1 (0.1)	76.3 (0.1)	76.4 (0.1)	.03
Female sex	37 185 (67.1)	9243 (65.9)	12 144 (67.5)	8605 (68.1)	7193 (67.1)	.01
White patients	41 978 (75.8)	9832 (70.1)	13 530 (75.2)	9939 (78.7)	8677 (80.9)	<.001
Rural location	6697 (12.1)	1430 (10.2)	2325 (12.9)	1598 (12.6)	1344 (12.5)	.09
Medicaid eligible	16 371 (29.6)	5446 (38.8)	5347 (29.7)	3247 (25.7)	2331 (21.7)	<.001
Household median income by zip code, mean (SE)	59910 (287)	58668 (569)	59423 (539)	60611 (561)	61528 (599)	.002
Elixhauser risk score, mean (SE)	3.91 (0.04)	4.21 (0.13)	3.89 (0.04)	3.81 (0.07)	3.68 (0.04)	<.001
Chronic condition indicators,						
Depression	19 773 (35.7)	5174 (36.9)	6364 (35.4)	4419 (35.0)	3816 (35.6)	.47
Substance use disorder	1214 (2.2)	419 (3.0)	363 (2.0)	260 (2.1)	172 (1.6)	.03
Anxiety disorders	11 969 (21.6)	3205 (22.8)	3908 (21.7)	2687 (21.3)	2169 (20.2)	.07
Opioid use disorders	545 (1.0)	162 (1.2)	178 (1.0)	120 (0.9)	85 (0.8)	.12

Abbreviations: MOC, maintenance of certification; SE, standard error.

^a^ Quartile 1 indicates the bottom quartile, and quartile 4 indicates the highest quartile.

### Opioid Prescription Rates and Association With Knowledge Quartile

Opioids were prescribed after 11 978 (21.6%) of LBP visits with 9759 (81.5%) of these prescriptions HDLD. The HDLD visit prescription rate was 17.6% (9759). Table 2 lists regression adjusted prescription rates by time and eTable 5 in the Supplement lists all regression coefficients. For visits with physicians in the highest knowledge quartile, there was a statistically significant 4.4 (95% CI, –6.8 to –2.0) percentage point decrease in adjusted prescribing comparing the early with late periods (*P* < .001). However, prescribing during visits with physicians in the lowest knowledge quartile did not change (0.7 percentage point increase; 95% CI, –2.3 to 3.7 percentage points; *P* = .65). Reflecting this, adjusted prescribing rates for visits with physicians in the highest and lowest knowledge quartiles were similar in the early period (difference 0.5 percentage points; 95% CI, –1.9 to 3.0 percentage points; (*P* = .68). However, by the late period, prescribing rates were 4.6 (95% CI, –7.5 to –1.8) percentage points lower for visits with physicians in the highest vs lowest knowledge quartile (*P* = .002), approximately a 20% lower prescribing rate comparing the highest with lowest quartile. The difference-in-differences estimated association between highest and lowest knowledge quartile in the early vs late time periods was a –5.1 (95%, CI –8.9 to –1.4) percentage point differential change over the study period (*P* = .008). This difference-in-differences estimate was similar when we compared the late period to the middle period (–4.1 [95% CI, –7.8 to –0.3 percentage points], *P* = .03).

**Table 2.** Association Between Knowledge Quartile and Opioid Prescribing by Early (2009-2011), Middle (2012-2014), and Late (2015-2017) Periods

Time	Knowledge quartile^a^													
	Unadjusted prescription rate, (95% CI)				Regression adjusted prescription rate, (95% CI)^b^				Percentage point difference, (95% CI)					
	1	2	3	4	1	2	3	4	2 vs 1	*P* value	3 vs 1	*P* value	4 vs 1	*P* value
**Any opioid prescription**														
Early	21.8 (19.2 to 24.3)	22.0 (20.5 to 23.6)	22.9 (21.2 to 24.5)	22.4 (20.5 to 24.4)	21.9 (20.1 to 23.7)	22.1 (20.8 to 23.4)	22.7 (21.3 to 24.2)	22.4 (20.8 to 24.1)	0.2 (–2.0 to 2.5)	.84	0.8 (–1.5 to 3.2)	.47	0.5 (–1.9 to 3.0)	.68
Middle	21.6 (19.1 to 24.1)	21.2 (19.5 to 22.8)	21.9 (20.2 to 23.5)	21.4 (19.7 to 23.1)	21.9 (20.1 to 23.8)	21.1 (19.6 to 22.6)	22.5 (21.0 to 24.0)	21.4 (19.8 to 23.1)	–0.8 (–3.2 to 1.5)	.49	0.6 (–1.8 to 3.0)	.64	–0.5 (–2.9 to 2.0)	.71
Late	22.1 (18.8 to 25.4)	21.7 (19.9 to 23.5)	20.2 (18.2 to 22.2)	18.3 (16.4 to 20.1)	22.6 (20.3 to 24.9)	20.9 (19.4 to 22.5)	19.5 (17.6 to 21.4)	18.0 (16.2 to 19.7)	–1.7 (–4.4 to 1.1)	.24	–3.1 (–6.0 to –0.1)	.04	–4.6 (–7.5 to –1.8)	.002
Knowledge quartile association differences middle vs early period	NA	NA	NA	NA	NA	NA	NA	NA	–1.1 (–4.4 to 2.2)	.52	–0.3 (–3.6 to 3.1)	.88	–1.0 (–4.5 to 2.5)	.58
Knowledge quartile association differences late vs middle period	NA	NA	NA	NA	NA	NA	NA	NA	–0.8 (–4.4 to 2.9)	.68	–3.6 (–7.3 to 0.2)	.06	–4.1 (–7.8 to –0.3)	.03
Knowledge quartile association differences late vs early period	NA	NA	NA	NA	NA	NA	NA	NA	–1.9 (–5.4 to 1.7)	.30	–3.9 (–7.7 to –0.1)	.04	–5.1 (–8.9 to –1.4)	.008
**High dosage or long duration opioid prescription**														
Early	18.4 (16.1 to 20.6)	18.0 (16.6 to 19.4)	17.8 (16.2 to 19.4)	17.3 (15.5 to 19.1)	17.8 (16.1 to 19.5)	17.8 (16.6 to 19.0)	17.9 (16.5 to 19.3)	17.7 (16.2 to 19.2)	0.1 (–2.0 to 2.1)	.95	0.2 (–2.0 to 2.3)	.89	–0.1 (–2.4 to 2.2)	.94
Middle	18.1 (15.8 to 20.3)	17.0 (15.6 to 18.3)	17.7 (16.2 to 19.2)	16.7 (15.2 to 18.2)	17.7 (16.0 to 19.4)	16.8 (15.5 to 18.1)	18.3 (16.9 to 19.7)	17.3 (15.9 to 18.8)	–0.9 (–3.1 to 1.2)	.39	0.6 (–1.6 to 2.8)	.61	–0.4 (–2.6 to 1.8)	.73
Late	19.1 (16.1 to 22.0)	19.2 (17.4 to 20.9)	16.4 (14.5 to 18.2)	13.9 (12.3 to 15.5)	19.2 (17.1 to 21.4)	18.3 (16.8 to 19.8)	16.4 (14.7 to 18.2)	14.5 (12.9 to 16.1)	–0.9 (–3.5 to 1.6)	.47	–2.8 (–5.5 to –0.1)	.04	–4.8 (–7.4 to –2.1)	<.001
Knowledge quartile association differences middle vs early period	NA	NA	NA	NA	NA	NA	NA	NA	–1.0 (–4.0 to 2.0)	.51	0.4 (–2.7 to 3.5)	.79	–0.3 (–3.5 to 2.9)	.85
Knowledge quartile association differences late vs middle period	NA	NA	NA	NA	NA	NA	NA	NA	0.1 (–3.3 to 3.4),	.98	–3.3 (–6.8 to 0.2)	.06	–4.3 (–7.7 to –0.9),	.01
Knowledge quartile association differences late vs early period	NA	NA	NA	NA	NA	NA	NA	NA	1.0 (–4.3 to 2.3)	.55	–3.0 (–6.5 to 0.6)	.10	–4.7 (–8.2 to –1.2)	.009
**Low dose short duration opioid prescription**														
Early	3.4 (2.7 to 4.0)	4.0 (3.5 to 4.6)	5.1 (4.3 to 5.8)	5.1 (4.3 to 5.9)	4.1 (3.4 to 4.8)	4.3 (3.7 to 4.9)	4.9 (4.2 to 5.6)	4.7 (4.0 to 5.4)	0.2 (–0.7 to 1.1)	.64	0.8 (–0.2 to 1.7)	.11	0.6 (–0.4 to 1.5)	.23
Middle	3.5 (2.8 to 4.3)	4.2 (3.6 to 4.9)	4.2 (3.4 to 5.0)	4.7 (3.9 to 5.6)	4.2 (3.4 to 5.0)	4.3 (3.7 to 4.9)	4.2 (3.5 to 4.9)	4.1 (3.4 to 4.8)	0.1 (–0.9 to 1.1)	.78	0.0 (–1.0 to 1.0)	1.00	–0.1 (–1.2 to 1.0)	.88
Late	3.0 (2.2 to 3.8)	2.6 (2.0 to 3.1)	3.8 (3.0 to 4.7)	4.4 (3.5 to 5.3)	3.3 (2.4 to 4.2)	2.6 (2.0 to 3.1)	3.2 (2.5 to 3.8)	3.3 (2.6 to 4.1)	–0.8 (–1.8 to 0.2)	.14	–0.2 (–1.2 to 0.9)	.77	0.0 (–1.1 to 1.1)	.98
Knowledge quartile association differences middle vs early period	NA	NA	NA	NA	NA	NA	NA	NA	–0.1 (–1.4 to 1.3)	.92	–0.8 (–2.2 to 0.6)	.28	–0.7 (–2.1 to 0.7)	.36
Knowledge quartile association differences late vs middle period	NA	NA	NA	NA	NA	NA	NA	NA	–0.9 (–2.3 to 0.5)	.20	–0.2 (–1.7 to 1.3)	.83	0.1 (–1.5 to 1.6)	.90
Knowledge quartile association differences late vs early period	NA	NA	NA	NA	NA	NA	NA	NA	–1.0 (–2.3 to 0.4)	.15	–0.9 (–2.4 to 0.5)	.20	–0.6 (–2.0 to 0.9)	.45

Abbreviation: NA, not applicable.

^a^ Quartile 1 indicates the bottom quartile, and quartile 4 indicates the highest quartile.

^b^ Regression adjusted means by quartile within year groups were constructed assuming a given quartile and year group interaction, holding all other covariates constant, and predicting prescription rates over the whole sample.

The late period knowledge associations were more strongly associated with HDLD than LDSD prescriptions. In this period, the adjusted HDLD prescription rate was 4.8 (95% CI, –7.2 to –2.1) percentage points lower during visits with physicians in the highest vs lowest knowledge quartiles, (*P* < .001), but was not statistically significant for LDSD prescriptions (0.0 [95% CI, –1.1 to 1.1 percentage points], *P* = .98). A simulation based on regression estimates indicated that if all clinically active outpatient internal medicine physicians prescribed opioids at the rate of physicians in the highest knowledge quartile, all else held equal, there would been 14 307 fewer opioid prescriptions for new concerns of LBP for Medicare fee-for-service Part D beneficiaries in the late period (Section 3 in the Supplement).

### Sensitivity Analyses

Alternative nonopioid pain medications were prescribed during LBP visits at similar rates across physician knowledge categories in the early period compared with the late period (regression adjusted prescription rates, 6.4% [95% CI, 5.5 to 6.7] vs 5.9% [95% CI, 5.1 to 6.7]), a difference of 0.4 percentage points (95% CI, –0.7 to 1.6 percentage points; *P* = .47) but diverged in the late period (eTable 4 in the Supplement). In the late period, the physicians in the lowest knowledge quartile prescribed recommended alternatives to opioids in fewer visits than physicians in the highest knowledge quartile (6.8% [95% CI, 5.7 to 7.9] vs 9.9% [95% CI, 8.4 to 11.5]), a difference of 3.2 percentage points (95% CI, 1.4 to 5.0 percentage points; *P* = .001). Our main results were robust to excluding physicians whose examinations occurred during the first half of 2008 when equated scores were derived, excluding physician training measures from the regression and modifying the middle and late periods to straddle the CDC guidelines changes in early 2016 ( eTable 6 to eTable 8 in the Supplement).

## Discussion

In this cross-sectional study, we found that from 2015 to 2017 (when awareness of the dangers of opioid overprescribing was increasing in medical literature and reflected in clinical guidelines), physicians with greater clinical knowledge, as measured by ABIM board examination performance, were less likely to prescribe opioids for the initial visit for older patients with new onset back pain than physicians with a lower performance. However, this difference was not observed during the earlier periods between 2009 to 2011. The magnitude of this association was clinically meaningful. For example, we found that during visits with physicians scoring in the highest vs lowest knowledge quartile opioid prescribing rates were similar during the early period (ie, 2009-2011) and middle period (ie, 2012-2014) compared with the late period, when this difference across knowledge quartiles was −4.6 percentage points, corresponding to approximately a 20% reduction comparing the highest with the lowest quartile. That this difference did not emerge until the late period suggests that it was associated with changes in guidelines, which began at the onset of this period. Furthermore, the reduction in opioid prescriptions among physicians with higher examination performance was focused on the most potentially excessive prescriptions, as indicated by differences in HDLD prescriptions. However, even in the late period, visits with physicians who scored in the highest knowledge quartile had opioid prescribing rates of approximately 18%, most of which likely did not conform to post-2015 clinical guidelines. To our knowledge, this reflects the continued challenge of opioid overprescribing and the relatively limited options for effective pain control in musculoskeletal care.^13,39^ In 2018, one-quarter of adults 65 years and older were prescribed an opioid.^10^

This is the first study to examine the differential association between physician knowledge and opioid prescribing during periods before and after opioid guidelines were changing. One study^40^ reported mixed evidence that the release of the 2016 CDC guidelines may have altered physician opioid prescribing behavior. Our sensitivity analysis suggested that opioid prescribing after this guideline change largely occurred among physicians who kept the most current with their medical knowledge, as reflected in examination performance, which partially explained these mixed findings of a reduction in opioid prescribing. This suggests that guideline changes need to be accompanied by education campaigns designed to reach physicians who are less diligent in keeping up with clinical guidelines. Other studies^41,42,43^ report a positive association between MOC examination performance and process-based quality measures and adverse outcomes at risk of diagnostic error. Better performance on ABIM examinations has also been found to be positively associated with higher residency ratings and participation in continuing medical education (CME) activities.^44,45^

Our findings suggest that performance on a knowledge examination is associated with clinically meaningful prescribing behavior. Physicians who score well on these examinations may be more responsive to changes in standards of care. However, continued prescribing of HDLD opioids by physicians who scored high on the MOC examination suggests that general knowledge-based interventions, although helpful, may not be sufficient to address opioid overprescribing. More targeted education interventions are also needed and may benefit from the involvement of both certifying and state licensure boards. Possible interventions include targeted CME requirements, or, for boards of certification or their medical societies, MOC modules related to emerging problems or direct communication with the physicians they serve.

### Limitations

This study had limitations. Our measures of opioid prescribing were based on Medicare fee-for-service beneficiaries older than 65 years with Part-D claims and may not generalize to other populations. Similarly, we picked a clinical scenario where opioids were likely to be inappropriate, and we cannot say whether the results we reported would be applicable to other clinical scenarios where opioids are also commonly prescribed. Because our measure of knowledge was for only a single point in time for a particular physician, we relied on serial cross-sections to infer associations with changes in physician prescribing over time. Furthermore, this measure of physician clinical knowledge was general in nature and not specifically targeted at opioid prescribing knowledge. However, back pain management is included as an examination component in ABIM’s internal medicine MOC examination blueprint. Furthermore, our goal was to measure the degree to which keeping current with general medical knowledge increases responsiveness to changes in guidelines or medical evidence of the kind examined in this study. Another limitation is that unobserved confounding between examination performance and opioid prescribing might have biased our results. We addressed this by accounting for a variety of practice characteristics and other measures that may be independently associated with opioid prescribing (eg, practicing in an academic medical center, practice size, rural location) as well as a variety of measures of patient demographic characteristics, health risk factors, and state opioid prescribing regulations. Controlling for physician training characteristics may have resulted in a bias toward the null in that these measures are likely also related to physician clinical knowledge. However, sensitivity analysis indicated that our results were robust to exclusion of these controls. Another limitation was that we could not directly account for physicians in the highest vs lowest knowledge quartile being more conservative in back pain management overall. However, we found that the physicians in the highest quartile were more likely to prescribe recommended alternatives to opioids. Although our results suggest that the types of opioid prescriptions most likely to be objectionable (HDLD prescriptions) were associated with differences in physicians’ knowledge, we were unable to directly observe the appropriateness of prescriptions.

## Conclusions

During the late 2015 to 2017 period of this cross-sectional study, the standard of care shifted away from the routine prescribing of opioids. Physicians who performed well on an ABIM examination assessing current knowledge were less likely to prescribe opioids for back pain than physicians who did not perform as well on the examination.

## Notes

Supplement. **eTable 1.** Low Back Pain ICD-9s/ICD-10s Inclusion and Exclusion Criteria **eAppendix 1.** Equated Examination Score Construction **eTable 2.** OLS Regression Used to Predict Equated Scores for First Examination in 2008 **eTable 3.** First Attempt Examination Score Quartile Breaks **eAppendix 2.** Policy Simulation **eAppendix 3.** Recommended Alternative Medication Sensitivity **eTable 4.** Association Between Knowledge Based on MOC Examination Performance Quartile and Recommended Alternative Prescribing by Periods **eTable 5.** Regression Odds Ratio and Summary Statistics **eAppendix 4.** Other Regression Sensitivities **eTable 6.** Regression Sensitivities for Any Opioids **eTable 7.** Regression Sensitivities for High Dosage and Long Duration **eTable 8.** Regression Sensitivities for Low Dosage or Short Duration eReferences.
