# Supplementary material for: Clinical Knowledge and Trends in Physicians' Prescribing of Opioids for New Onset Back Pain, 2009-2017
Source: JAMA Netw Open. 2021 Jul 1;4(7):e2115328. doi: 10.1001/jamanetworkopen.2021.15328 (PMC8251502; doi:10.1001/jamanetworkopen.2021.15328)
Supplement: Supplement. — eTable 1. Low Back Pain ICD-9s/ICD-10s Inclusion and Exclusion Criteria eAppendix 1. Equated Examination Score Construction eTable 2. OLS Regression Used to Predict Equated Scores for First Examination in 2008 eTable 3. First Attempt Examination Score Quartile Breaks eAppendix 2. Policy Simulation eAppendix 3. Recommended Alternative Medication Sensitivity eTable 4. Association Between Knowledge Based on MOC Examination Performance Quartile and Recommended Alternative Prescribing by Periods eTable 5. Regression Odds Ratio and Summary Statistics eAppendix 4. Other Regression Sensitivities eTable 6. Regression Sensitivities for Any Opioids eTable 7. Regression Sensitivities for High Dosage and Long Duration eTable 8. Regression Sensitivities for Low Dosage or Short Duration eReferences. [file jamanetwopen-e2115328-s001.pdf]

Supplemental Online Content

Gray BM, Vandergrift JL, Weng W, Lipner RS, Barnett ML. Clinical knowledge and trends in physicians' prescribing of opioids for new onset back pain, 2009-2017. *JAMA Netw Open*. 2021;4(7):e2115328. doi:10.1001/jamanetworkopen.2021.15328

eTable 1. Low Back Pain ICD-9s/ICD-10s Inclusion and Exclusion Criteria

eAppendix 1. Equated Examination Score Construction

eTable 2. OLS Regression Used to Predict Equated Scores for First Examination in 2008

eTable 3. First Attempt Examination Score Quartile Breaks

eAppendix 2. Policy Simulation

eAppendix 3. Recommended Alternative Medication Sensitivity

eTable 4. Association Between Knowledge Based on MOC Examination Performance Quartile and Recommended Alternative Prescribing by Periods

eTable 5. Regression Odds Ratio and Summary Statistics

eAppendix 4. Other Regression Sensitivities

eTable 6. Regression Sensitivities for Any Opioids

eTable 7. Regression Sensitivities for High Dosage and Long Duration

eTable 8. Regression Sensitivities for Low Dosage or Short Duration

eReferences.

This supplemental material has been provided by the authors to give readers additional information about their work.

© 2021 Gray BM et al. *JAMA Network Open*.

eTable 1. ICD-9 and ICD-10 Codes for Back Pain Inclusion and Exclusion Criteria[1]

ICD-9 code	ICD-10	Description
Inclusion Criteria		
721.3, 722.10, 722.52, 722.73, 722.93, 724.02, 724.2, 724.3, 724.4, 724.5, 756.11, 756.12, 846.0, 846.1, 846.8, 846.9, 847.2, 847.3	M47.817, M51.21, M51.27, M51.36, M51.37, M51.06, M51.07, M46.47, M51.86, M51.87, M48.06, M54.5, M54.30, M54.14 – M54.17, M54.89, M54.9, Q76.2, S33.8XXA, S33.6XXA, S33.8XXA, S33.9XXA, S33.5XXA, S33.8XXA	Low Back Pain
Exclusion Criteria		
592	N20.0, N20.1, N20.9	Calculus of kidney
574.20	K80.20	Calculus of gallbladder without mention of cholecystitis
599.0	N39.0	Urinary tract infection, site not specified
V13.02	Z87.440	Urinary (tract) infection
140 – 239	C00 – D49	Neoplasms
V17.81, V82.81	Z82.62, Z13.820	Osteoporosis
344.6	G83.4	Cauda equine syndrome
730	M86.xx, M89.60, M89.619, M89.629, M89.639, M89.649, M89.659, M89.669, M89.679, M89.968, M89.69, M90.80, M90.810, M90.829, M90.830, M90.849, M90.859, M90.869, M90.879, M90.88, M90.89, M46.20, M46.30	Osteomyelitis, periostitis, and other bone infections
731.3	M89.70	Major osseous deficit

© 2021 Gray BM et al. *JAMA Network Open*.

eAppendix 1. Equated Examination Score Construction

ABIM and other certifying boards typically create and administer parallel forms of an exam with different questions because of item exposure and test security issues. Although these parallel forms are constructed to be as similar as possible in terms of content and statistical specifications, they may slightly differ in difficulty. As a result, small adjustments are made to correct for differences in difficulty in order for examinee scores to be exactly comparable across adjacent forms allowing for fair pass fail decisions. This adjustment process is known as "equating". As such, equating adjusts for differences in difficulty among forms that are built to be similar in difficulty and content."[2]

Starting in the second exam administration in 2008, ABIM's psychometric department began equating exam scores using Item Response Theory (IRT). IRT is a process by which exam scores are equated by comparing how examinees perform on identical questions across exam administration.[3] Differences in performance on these questions and questions that were not identical across exam forms are then used to adjust scores so that the final score allows for fair comparisons across forms. This equated also incorporate other information used for equating related to performance of similar groups of physicians, such as physicians with the same residency training program and similar evaluations who presumably would score similarly on an examination.

Formal IRT adjustment is fairly involved so for the first exam administration in 2008, since IRT equated scores stated after that period, we constructed equated scores by regressing IRT equated scores from all other exams against variables correlated with underlying clinical knowledge, the pass/fail indicator as well as the raw score itself. Using this regression model, we then predicted equated scores for the initial 2008 exam administration (see eTable 2 for the regression statistics and explanatory variables). Notably this regression explained over 90% of the variance in equated scores.

© 2021 Gray BM et al. *JAMA Network Open*.

eTable 2. OLS Regression Used to Predict Equated Scores for First Examination in 2008

Number of observations: 27,843<br>F(10, 27,843) = 32,529.8, Prob > F = 0<br>R-squared=92.12 Adjusted=92.12<br>Root MSE = 28.488			
	Coefficient	SE	P-stat
Exam performance percentile	11.55	0.03	<.001
Physician Age	-0.04	0.03	0.24
Physician Female	-1.80	0.35	<.001
Internal medical school graduate (IMG) versus non-DO US medical school graduate	3.96	1.62	0.01
DO non-DO US medical school graduate	2.00	1.67	0.23
US birth	-2.60	1.82	0.15
US birth interacted with IMG medical school Graduate	-0.75	2.03	0.71
US birth interacted with DO indicator	0.59	1.91	0.76
Failed MOC exam	10.95	0.73	<.001
MOC exam year	6.60	0.07	<.001
Constant	-13656.8	143.17	<.001

eTable 3. First Attempt Examination Score Quartile Breaks

	Equated score percentile breaks		
Exam Year	25th	50th	75th
2008	421	492	561
2009	415	485	563
2010	407	481	556
2011	402	474	549
2012	392	464	540
2013	371	442	517
2014	371	444	519
2015	410	479	553
2016	419	487	559

© 2021 Gray BM et al. *JAMA Network Open*.

eAppendix 2. Policy Simulation

We conducted a policy simulation that applied the results of our estimation of opioid prescribing rates. In this simulation we estimated the difference in the number of opioid prescription during new LBP visits for Medicare fee-for-service beneficiaries with Part-D coverage resulting in an opioid prescription written by all clinically active US general internists. We first simulated the predicted visit level probability of an opioid prescription by apply the coefficient estimates in combination with the actually visit, patient and physicians characteristics for the covariate characteristics in the regression model. We then summed this figure across all visits during the 2015 to 2017 period. This figure is prescriptions **2,816**; (mean visit prescription rate 20.8%). Next we ran this same simulation but set the knowledge quartile level to 4 across the whole sample of visits (including year group interaction terms and year indicators; prescription rate=18.5%). We then summed this figure across all visits during the 2015 to 2017 period yielding the estimated number of opioid prescriptions if every physician in the sample during the 2015 to 2017 period had scored in the top exam quartile. For this simulation the estimated number of prescriptions during this period is **2,503**. The difference between these two figures (**313**) yield the potential for improvement in opioid prescription estimates for knowledge quartile within the sample of physician we studied. We scaled this number to the population level by first dividing these figures by the total number of active physician in the sample yearly (**2,681**) which yields a rate of opioid prescribed and then multiplying this number by the population level 2019 estimates of the total number of active physicians of 120,171 (this assumes that change in the total number of active physicians between 2015 and 2019 were not material). Based on these calculations the predicted actually number of prescriptions during the 2015 to 2017 period assuming actually characteristics and scaled to the population level is estimated to be 126,058 (**128,717**=1.07*120,171). Assuming every physician scored in the top exam quartile this figure is 112,047 (**114,410**=0.95*120,171) or a **14,307** reduction in opioid prescribing.

© 2021 Gray BM et al. *JAMA Network Open*.

eAppendix 3. Recommended Alternative Medication Sensitivity

Recommended alternative medications for established back pain that were not available over the counter and were appropriate for older age populations were applied in the study.[4-6] These recommended alternatives included anticonvulsive, antidepressants, topical pharmacologic agents and memantine; but excluded non-steroidal anti-inflammatory drugs (NSAIDs) and acetaminophen, because they are available over the counter, and benzodiazepines and tricyclic antidepressants (TCA) because they are potentially inappropriate medications to prescribe to older age populations per the AGS Beers criteria guidelines.[7]

eTable 4 Association Between Knowledge Quartile Based on MOC Examination Performance Quartile and Recommended Alternative Prescribing by Periods

(2009-2011, 2012-2014, 2015-2017)

	Regression adjusted mean percent by knowledge quartile, (95% CI)				Percentage point difference compared to quartile 1, 95% CI, P-value					
	Quartile 1 (bottom)	Quartile 2	Quartile 3	Quartile 4 (top)	Quartile 2	P	Quartile 3	P	Quartile 4	P
Year										
2009-2011	5.9 (5.1 to 6.7)	7.0 (6.1 to 7.8)	6.3 (5.5 to 7.1)	6.4 (5.5 to 7.2)	1.0 (-0.1 to 2.2)	0.07	0.4 (-0.7 to 1.5)	0.52	0.4 (-0.7 to 1.6)	0.47
2012-2014	8.4 (7.0 to 9.8)	7.9 (7.1 to 8.7)	9.0 (7.9 to 10.1)	8.5 (7.3 to 9.7)	-0.5 (-2.1 to 1.1)	0.53	0.6 (-1.2 to 2.4)	0.50	0.1 (-1.7 to 1.9)	0.88
2015-2017	6.8 (5.7 to 7.9)	8.2 (7.1 to 9.3)	8.9 (7.6 to 10.3)	9.9 (8.4 to 11.5)	1.4 (-0.1 to 2.9)	0.07	2.2 (0.5 to 3.8)	0.01	3.2 (1.4 to 5.0)	0.001
Quartile difference comparing 2012-2014 to 2009-2011					-1.5 (-3.5 to 0.4)	0.12	0.3 (-1.8 to 2.3)	0.80	-0.3 (-2.4 to 1.8)	0.79
Quartile difference comparing 2015-2017 to 2012-2014					1.9 (-0.3 to 4.1)	0.08	1.6 (-0.9 to 4.0)	0.21	3.0 (0.5 to 5.6)	0.02
Quartile difference comparing 2015-2017 to 2009-2011					0.4 (-1.5 to 2.2)	0.69	1.8 (-0.2 to 3.8)	0.08	2.7 (0.6 to 4.9)	0.01

eTable 5: Regression Odds Ratio and Summary Statistics

	Any Opioid		High dosage or long duration		Low dosage and short duration	
	Wald chi2(153)=1606.95, P<0.001		Wald chi2(153)=1757.94, P<0.001		Wald chi2(153)=675.5, P<0.001	
	OR (SE)	P	OR (SE)	P	OR (SE)	P
Knowledge (i.e,, MOC exam score) quartile (Q)						
Q1 - Bottom	ref		ref		ref	
Q2	1.01 (0.07)	0.87	1.00 (0.08)	0.98	1.05 (0.12)	0.66
Q3	1.05 (0.07)	0.52	1.01 (0.08)	0.94	1.20 (0.14)	0.11
Q4 - Top	1.03 (0.08)	0.73	0.99 (0.08)	0.90	1.15 (0.13)	0.24
Time Period						
2009-2011	ref		ref		ref	
2012-2014	1.03 (0.10)	0.77	1.03 (0.10)	0.77	0.99 (0.16)	0.95
2015-2017	0.91 (0.10)	0.38	0.95 (0.11)	0.67	0.77 (0.18)	0.26
Knowledge quartile interacted (X) Year Group Interaction						
Q2 X 2012-2014	0.94 (0.10)	0.53	0.93 (0.10)	0.52	0.99 (0.17)	0.97
Q2 X 2015-2017	0.86 (0.09)	0.16	0.90 (0.11)	0.35	0.69 (0.14)	0.07
Q3 X 2012-2014	0.98 (0.10)	0.88	1.03 (0.12)	0.80	0.84 (0.15)	0.33
Q3 X 2015-2017	0.76 (0.09)	0.02	0.78 (0.10)	0.06	0.77 (0.16)	0.19
Q4 X 2012-2014	0.94 (0.10)	0.59	0.98 (0.12)	0.85	0.87 (0.16)	0.43
Q4 X 2015-2017	0.71 (0.09)	0.01	0.70 (0.09)	0.01	0.87 (0.18)	0.51
Year						
2009	Reference for period for year 2009-2011					
2010	1.08 (0.06)	0.16	1.06 (0.06)	0.32	1.10 (0.10)	0.29
2011	1.02 (0.06)	0.81	0.98 (0.07)	0.74	1.14 (0.11)	0.20
2012	1.07 (0.07)	0.26	1.03 (0.07)	0.68	1.22 (0.13)	0.07
2013	0.94 (0.06)	0.31	0.91 (0.06)	0.19	1.07 (0.12)	0.52
2014	Reference for period for years 2012-2014					
2015	1.36 (0.11)	<.001	1.34 (0.12)	0.00	1.26 (0.21)	0.16
2016	1.20 (0.10)	0.03	1.21 (0.11)	0.04	1.07 (0.17)	0.67
2017	Reference for period for years 2015-2017					
Month						
January	ref		ref		ref	
February	1.04 (0.06)	0.44	1.05 (0.06)	0.38	0.99 (0.11)	0.93
March	0.94 (0.05)	0.25	0.93 (0.05)	0.20	1.01 (0.11)	0.94
April	0.97 (0.05)	0.57	0.96 (0.05)	0.49	1.01 (0.11)	0.92
May	0.92 (0.05)	0.12	0.91 (0.05)	0.11	0.98 (0.10)	0.87
June	0.99 (0.05)	0.81	0.99 (0.06)	0.83	0.98 (0.11)	0.88
July	0.93 (0.05)	0.15	0.93 (0.05)	0.21	0.93 (0.10)	0.53
August	0.92 (0.05)	0.12	0.93 (0.05)	0.19	0.93 (0.10)	0.49
September	1.01 (0.05)	0.88	0.98 (0.06)	0.69	1.12 (0.12)	0.30
October	0.96 (0.05)	0.46	0.95 (0.05)	0.36	1.03 (0.11)	0.79
November	0.93 (0.05)	0.18	0.93 (0.05)	0.20	0.96 (0.11)	0.71
December	1.04 (0.06)	0.51	1.04 (0.06)	0.50	1.00 (0.11)	1.00
Female Physician	0.88 (0.03)	0.00	0.88 (0.04)	0.00	0.95 (0.05)	0.35
International medical school	1.14 (0.07)	0.02	1.18 (0.08)	0.01	1.05 (0.10)	0.61
International birth	1.05 (0.06)	0.36	1.11 (0.07)	0.09	0.90 (0.08)	0.25
International medical school and Birth	0.98 (0.08)	0.81	1.02 (0.09)	0.86	0.77 (0.10)	0.05
Type of practice						
Group Practice	ref		ref		ref	
Community Health Center	1.44 (0.24)	0.03	1.57 (0.28)	0.01	0.94 (0.29)	0.85
Nursing Home	1.35 (0.13)	0.00	1.40 (0.14)	0.00	1.00 (0.16)	0.99
VA/Military/Government	1.06 (0.15)	0.65	1.09 (0.17)	0.56	0.96 (0.24)	0.88
Academic practice	0.79 (0.06)	0.00	0.87 (0.07)	0.07	0.62 (0.08)	<.001
Other	1.10 (0.20)	0.60	0.84 (0.17)	0.38	2.12 (0.57)	0.01
Completed a legacy MOC survey	0.93 (0.04)	0.06	0.95 (0.04)	0.19	0.88 (0.06)	0.07
Practice Size (number of physicians)						
1	ref		ref		ref	
2 to 5	0.94 (0.04)	0.13	0.89 (0.04)	0.01	1.21 (0.10)	0.02
6 to 10	0.98 (0.05)	0.74	0.94 (0.05)	0.28	1.23 (0.11)	0.03
11 to 15	0.98 (0.07)	0.72	0.85 (0.06)	0.03	1.56 (0.19)	<.001

© 2021 Gray BM et al. *JAMA Network Open*.

16 to 25	0.95 (0.07)	0.47	0.90 (0.07)	0.18	1.26 (0.16)	0.08
26 to 50	0.85 (0.07)	0.03	0.86 (0.07)	0.07	0.93 (0.14)	0.63
>=50	0.98 (0.06)	0.73	0.91 (0.06)	0.15	1.32 (0.14)	0.01
Unknown	0.69 (0.10)	0.01	0.70 (0.12)	0.04	0.78 (0.24)	0.42
Number of Physician	1.09 (0.23)	0.69	1.10 (0.26)	0.70	1.05 (0.43)	0.90
number of visits	1.00 (0.00)	0.93	1.00 (0.00)	0.20	1.00 (0.00)	0.00
number of visits squared	1.00 (0.00)	0.18	1.00 (0.00)	0.05	1.00 (0.00)	0.39
Beneficiary Age	0.95 (0.03)	0.07	0.96 (0.03)	0.20	0.93 (0.06)	0.23
Beneficiary Age squared	1.00 (0.00)	0.11	1.00 (0.00)	0.25	1.00 (0.00)	0.27
Beneficiary Race						
White	ref		ref		ref	
Black	1.01 (0.05)	0.91	1.07 (0.05)	0.18	0.76 (0.08)	0.01
Hispanic	0.81 (0.05)	0.00	0.81 (0.05)	0.00	0.87 (0.10)	0.26
Asian	0.53 (0.05)	<.001	0.53 (0.05)	<.001	0.67 (0.11)	0.01
Other/Unknown	0.71 (0.07)	<.001	0.73 (0.07)	0.00	0.70 (0.15)	0.10
Beneficiary female	0.97 (0.03)	0.45	0.94 (0.04)	0.08	1.14 (0.08)	0.05
Rural Location	1.10 (0.05)	0.02	1.15 (0.05)	0.00	0.92 (0.07)	0.29
Medicaid eligible	1.16 (0.04)	<.001	1.26 (0.05)	<.001	0.72 (0.05)	<.001
Household median income (per $10k)	1.00 (0.00)	<.001	1.00 (0.00)	<.001	1.00 (0.00)	0.53
Prior Health contacts/care						
ED visit <14 days prior	1.23 (0.10)	0.01	1.16 (0.11)	0.11	1.43 (0.24)	0.04
Hospitalization <14 day prior	0.80 (0.16)	0.27	0.82 (0.17)	0.34	0.81 (0.40)	0.67
ED visit <30 day prior	0.96 (0.08)	0.61	0.96 (0.08)	0.60	1.00 (0.17)	0.99
Surgery <30 day prior	1.08 (0.23)	0.73	0.98 (0.22)	0.94	1.63 (0.81)	0.32
Hospitalization <30 day prior	0.97 (0.16)	0.85	1.03 (0.18)	0.89	0.73 (0.29)	0.43
ED visit <90 day prior	1.05 (0.06)	0.40	1.06 (0.06)	0.34	0.99 (0.12)	0.93
Surgery <90 day prior	1.16 (0.15)	0.23	1.14 (0.15)	0.31	1.18 (0.36)	0.59
Hospitalization <90d prior	0.93 (0.10)	0.52	0.94 (0.11)	0.58	0.94 (0.24)	0.82
ED visit <80 day prior	1.09 (0.05)	0.05	1.09 (0.05)	0.07	1.06 (0.10)	0.52
Surgery <180 day prior	0.97 (0.08)	0.71	1.02 (0.09)	0.86	0.79 (0.17)	0.28
Hospitalization <180 day prior	1.12 (0.08)	0.13	1.13 (0.09)	0.12	1.02 (0.18)	0.93
Elixhauser risk score	1.01 (0.01)	0.26	1.01 (0.01)	0.09	0.98 (0.01)	0.18
Chronic Condition indicators						
Alzheimer's Disease and Related Disorders or Senile Dementia	0.90 (0.04)	0.01	0.90 (0.04)	0.02	0.94 (0.09)	0.47
Alzheimer's Disease	0.91 (0.06)	0.15	0.95 (0.06)	0.48	0.71 (0.11)	0.03
Acute Myocardial Infarction	1.04 (0.06)	0.45	1.04 (0.06)	0.55	1.05 (0.12)	0.63
Anemia	1.01 (0.03)	0.73	1.02 (0.03)	0.48	0.97 (0.05)	0.61
Asthma	0.98 (0.03)	0.44	0.98 (0.03)	0.49	0.97 (0.07)	0.66
Atrial Fibrillation	1.11 (0.04)	0.00	1.08 (0.04)	0.02	1.20 (0.08)	0.01
Breast cancer	1.00 (0.04)	0.98	1.03 (0.05)	0.53	0.91 (0.09)	0.30
Colorectal Cancer	1.05 (0.07)	0.43	1.06 (0.07)	0.42	1.01 (0.14)	0.92
Endometrial Cancer	1.04 (0.11)	0.73	1.06 (0.12)	0.61	0.93 (0.21)	0.74
Lung Cancer	1.21 (0.11)	0.04	1.17 (0.12)	0.13	1.31 (0.24)	0.15
Prostate Cancer	0.96 (0.05)	0.47	0.92 (0.06)	0.17	1.14 (0.12)	0.23
Cataract	0.90 (0.02)	<.001	0.90 (0.03)	<.001	0.96 (0.05)	0.49
Heart Failure	1.07 (0.03)	0.02	1.08 (0.03)	0.02	1.01 (0.06)	0.90
Chronic Kidney Disease	1.15 (0.03)	<.001	1.16 (0.04)	<.001	1.04 (0.06)	0.52
Chronic Obstructive Pulmonary Disease	1.24 (0.03)	<.001	1.27 (0.04)	<.001	1.04 (0.06)	0.55
Depression	1.04 (0.03)	0.11	1.07 (0.03)	0.01	0.93 (0.05)	0.18
Diabetes	1.06 (0.03)	0.02	1.05 (0.03)	0.07	1.07 (0.06)	0.17
Glaucoma	0.93 (0.02)	0.01	0.90 (0.03)	<.001	1.05 (0.05)	0.31
Hip/Pelvic Fracture	1.31 (0.08)	<.001	1.32 (0.09)	<.001	1.15 (0.15)	0.28
Hyperlipidemia	0.90 (0.03)	0.00	0.90 (0.03)	0.00	0.96 (0.06)	0.57
Benign Prostatic Hyperplasia	0.83 (0.03)	<.001	0.81 (0.04)	<.001	0.97 (0.08)	0.72
Hypertension	1.34 (0.05)	<.001	1.36 (0.06)	<.001	1.21 (0.08)	0.01
Hypothyroidism	0.90 (0.02)	<.001	0.91 (0.03)	0.00	0.91 (0.05)	0.09
Ischemic Heart Disease	0.98 (0.03)	0.53	0.98 (0.03)	0.42	1.01 (0.06)	0.85
Osteoporosis	0.94 (0.03)	0.02	0.95 (0.03)	0.09	0.90 (0.05)	0.06
Rheumatoid Arthritis	1.17 (0.03)	<.001	1.25 (0.04)	<.001	0.89 (0.05)	0.02
Stroke	0.97 (0.03)	0.38	0.95 (0.03)	0.12	1.08 (0.07)	0.24
Anxiety	1.11 (0.03)	<.001	1.09 (0.03)	0.01	1.15 (0.07)	0.02
Opioid use disorder	1.61 (0.17)	<.001	1.75 (0.20)	<.001	0.70 (0.21)	0.23
Substance use disorders	1.31 (0.11)	0.00	1.30 (0.12)	0.00	1.11 (0.21)	0.59
State Opioid Policies						

© 2021 Gray BM et al. *JAMA Network Open*.

State opioid prescription registration requited	1.09 (0.08)	0.27	1.07 (0.09)	0.42	1.16 (0.16)	0.27
State opioid dosage/duration regulation	0.93 (0.11)	0.52	1.01 (0.13)	0.91	0.73 (0.14)	0.11
Having both state opioid registration requirement and opioid regulations	0.71 (0.13)	0.06	0.54 (0.11)	0.00	1.88 (0.52)	0.02
Beneficiary State						
Alabama	ref		ref		ref	
Alaska	0.86 (1.00)	0.89	0.35 (0.34)	0.28	2.26 (2.57)	0.47
Arizona	0.71 (0.10)	0.01	0.80 (0.12)	0.13	0.49 (0.14)	0.01
Arkansas	0.76 (0.16)	0.19	0.81 (0.19)	0.36	0.68 (0.27)	0.34
California	0.65 (0.08)	0.00	0.68 (0.09)	0.00	0.65 (0.14)	0.04
Colorado	0.63 (0.12)	0.01	0.63 (0.12)	0.02	0.75 (0.22)	0.32
Connecticut	0.57 (0.08)	<.001	0.55 (0.09)	<.001	0.78 (0.20)	0.32
Delaware	0.57 (0.16)	0.04	0.57 (0.18)	0.07	0.72 (0.34)	0.49
District of Columbia	0.38 (0.12)	0.00	0.34 (0.11)	0.00	0.81 (0.48)	0.72
Florida	0.60 (0.07)	<.001	0.65 (0.09)	0.00	0.51 (0.11)	0.00
Georgia	0.98 (0.13)	0.88	0.92 (0.13)	0.56	1.21 (0.26)	0.40
Hawaii	0.66 (0.16)	0.08	0.82 (0.22)	0.47	0.33 (0.19)	0.06
Idaho	0.55 (0.15)	0.03	0.46 (0.13)	0.01	1.02 (0.44)	0.96
Illinois	0.65 (0.09)	0.00	0.61 (0.09)	0.00	0.95 (0.21)	0.83
Indiana	0.92 (0.15)	0.60	0.93 (0.15)	0.68	0.88 (0.23)	0.61
Iowa	0.84 (0.15)	0.34	0.80 (0.16)	0.25	1.03 (0.35)	0.92
Kansas	0.88 (0.17)	0.52	0.83 (0.19)	0.40	1.10 (0.38)	0.77
Kentucky	1.09 (0.19)	0.60	1.23 (0.21)	0.23	0.57 (0.16)	0.04
Louisiana	1.01 (0.17)	0.95	0.97 (0.16)	0.86	1.11 (0.33)	0.73
Maine	0.55 (0.13)	0.01	0.57 (0.15)	0.03	0.66 (0.24)	0.25
Maryland	0.66 (0.09)	0.00	0.64 (0.09)	0.00	0.86 (0.22)	0.55
Massachusetts	0.68 (0.09)	0.00	0.68 (0.10)	0.01	0.75 (0.17)	0.19
Michigan	0.80 (0.10)	0.09	0.90 (0.12)	0.42	0.51 (0.12)	0.00
Minnesota	0.79 (0.13)	0.16	0.68 (0.13)	0.04	1.16 (0.30)	0.58
Mississippi	0.79 (0.14)	0.17	0.81 (0.14)	0.21	0.77 (0.26)	0.45
Missouri	0.89 (0.16)	0.51	0.79 (0.15)	0.22	1.33 (0.42)	0.37
Montana	0.87 (0.19)	0.52	0.72 (0.20)	0.24	1.32 (0.51)	0.47
Nebraska	0.84 (0.15)	0.35	0.64 (0.12)	0.02	1.57 (0.49)	0.15
Nevada	0.83 (0.13)	0.23	0.97 (0.17)	0.85	0.29 (0.18)	0.05
New Hampshire	0.62 (0.12)	0.01	0.61 (0.12)	0.01	0.78 (0.26)	0.46
New Jersey	0.53 (0.07)	<.001	0.53 (0.07)	<.001	0.68 (0.15)	0.08
New Mexico	0.59 (0.14)	0.03	0.55 (0.14)	0.02	0.88 (0.38)	0.77
New York	0.28 (0.04)	<.001	0.30 (0.05)	<.001	0.31 (0.07)	<.001
North Carolina	0.86 (0.11)	0.23	0.93 (0.13)	0.59	0.66 (0.15)	0.07
North Dakota	0.61 (0.20)	0.13	0.72 (0.25)	0.35	0.38 (0.23)	0.10
Ohio	0.76 (0.10)	0.04	0.75 (0.11)	0.04	0.88 (0.20)	0.57
Oklahoma	0.91 (0.22)	0.69	0.99 (0.22)	0.95	0.65 (0.28)	0.31
Oregon	0.85 (0.12)	0.25	0.80 (0.13)	0.17	1.00 (0.25)	1.00
Pennsylvania	0.47 (0.07)	<.001	0.48 (0.08)	<.001	0.56 (0.13)	0.01
Rhode Island	0.69 (0.18)	0.15	0.55 (0.12)	0.01	1.38 (0.59)	0.46
South Carolina	0.78 (0.13)	0.13	0.82 (0.15)	0.27	0.72 (0.19)	0.21
South Dakota	0.74 (0.23)	0.34	0.55 (0.25)	0.19	1.48 (0.63)	0.36
Tennessee	1.04 (0.15)	0.76	1.11 (0.17)	0.47	0.77 (0.22)	0.36
Texas	0.75 (0.09)	0.02	0.73 (0.10)	0.02	0.93 (0.19)	0.73
Utah	0.94 (0.19)	0.76	0.98 (0.20)	0.91	0.84 (0.32)	0.65
Vermont	0.57 (0.14)	0.02	0.40 (0.13)	0.01	1.39 (0.49)	0.35
Virginia	0.83 (0.12)	0.20	0.82 (0.12)	0.19	0.94 (0.22)	0.79
Washington	0.95 (0.14)	0.73	0.93 (0.15)	0.66	1.01 (0.24)	0.96
West Virginia	0.88 (0.18)	0.54	0.92 (0.20)	0.68	0.77 (0.31)	0.52
Wisconsin	0.92 (0.13)	0.57	0.75 (0.11)	0.05	1.51 (0.35)	0.07
Wyoming	0.60 (0.18)	0.08	0.64 (0.21)	0.18	0.61 (0.46)	0.51
Constant	3.49 (4.06)	0.28	1.44 (1.80)	0.77	1.68 (4.13)	0.83

© 2021 Gray BM et al. *JAMA Network Open*.

eAppendix 4. Other Regression Sensitivities

Sensitivity analyses included: 1) Center for Disease Control (CDC) guideline change: adjust timing between middle period and last period to before and after CDC opioid guideline changes in April 2016, 2) excluding exams with regression adjusted equated scores (regression N = 51,814), and 3) excluding training controls (internal medical school interacted with internal birth).

© 2021 Gray BM et al. *JAMA Network Open*.

|eTable 6. Regression Sensitivities for Any Opioids

Years	Sensitivity Type	Regression adjusted mean percent by knowledge quartile, (95% CI)[a]				Percentage point difference compared to quartile 1, 95% CI, P-value		
		Quartile 1 (bottom)	Quartile 2	Quartile 3	Quartile 4 (top)	Quartile 2	Quartile 3	Quartile 4
2009-2011	Base	21.9 (20.1 to 23.7)	22.1 (20.8 to 23.4)	22.7 (21.3 to 24.2)	22.4 (20.8 to 24.1)	0.2 (-2.0 to 2.5), P=0.84	0.8 (-1.5 to 3.2), P=0.47	0.5 (-1.9 to 3.0), P=0.68
	CDC guideline change	21.9 (20.1 to 23.7)	22.1 (20.8 to 23.4)	22.7 (21.3 to 24.2)	22.4 (20.8 to 24.1)	0.2 (-2.0 to 2.5), P=0.84	0.8 (-1.5 to 3.2), P=0.47	0.5 (-1.9 to 3.0), P=0.68
	Exclude the 1st Exam administration in 2008	21.9 (19.8 to 24.1)	22.2 (20.8 to 23.6)	23.3 (21.7 to 24.9)	22.0 (20.2 to 23.7)	0.3 (-2.3 to 2.8), P=0.83	1.3 (-1.3 to 4.0), P=0.32	0.0 (-2.7 to 2.8), P=0.98
	No training regression controls	21.8 (20.0 to 23.7)	22.1 (20.8 to 23.4)	22.5 (21.1 to 24.0)	22.0 (20.4 to 23.7)	0.3 (-1.9 to 2.5), P=0.79	0.7 (-1.6 to 3.0), P=0.56	0.2 (-2.2 to 2.6), P=0.88
2012-2014[b]	Base	21.9 (20.1 to 23.8)	21.1 (19.6 to 22.6)	22.5 (21.0 to 24.0)	21.4 (19.8 to 23.1)	-0.8 (-3.2 to 1.5), P=0.49	0.6 (-1.8 to 3.0), P=0.64	-0.5 (-2.9 to 2.0), P=0.71
	CDC guideline change[b]	22.6 (20.5 to 24.8)	19.4 (18.1 to 20.7)	21.6 (20.2 to 23.0)	23.4 (21.7 to 25.1)	-1.1 (-3.1 to 0.9), P=0.27	-0.1 (-2.2 to 1.9), P=0.89	-1.1 (-3.2 to 1.0), P=0.32
	Exclude the 1st Exam administration in 2008	21.9 (20.0 to 23.8)	21.1 (19.6 to 22.5)	22.5 (21.0 to 24.0)	21.5 (19.9 to 23.2)	-0.8 (-3.2 to 1.6), P=0.49	0.6 (-1.8 to 3.0), P=0.63	-0.3 (-2.8 to 2.1), P=0.79
	No training regression controls	22.0 (20.0 to 23.9)	21.1 (19.6 to 22.5)	22.4 (20.9 to 23.9)	21.3 (19.7 to 22.9)	-0.9 (-3.3 to 1.5), P=0.46	0.5 (-2.0 to 2.9), P=0.71	-0.7 (-3.2 to 1.8), P=0.60
						Quartile difference comparing 2012-2014 to 2009-2011		
	Base					-1.1 (-4.4 to 2.2), P=0.52	-0.3 (-3.6 to 3.1), P=0.88	-1.0 (-4.5 to 2.5), P=0.58
	CDC guideline change[b]					-1.1 (-4.0 to 1.8), P=0.46	-1.2 (-4.3 to 1.9), P=0.44	-1.9 (-5.1 to 1.3), P=0.25
	Exclude the 1st Exam administration in 2008					-1.1 (-4.6 to 2.4), P=0.53	-0.7 (-4.3 to 2.9), P=0.69	-0.4 (-4.1 to 3.3), P=0.84
	No training regression controls					-1.2 (-4.5 to 2.1), P=0.47	-0.2 (-3.6 to 3.1), P=0.90	-0.9 (-4.4 to 2.6), P=0.63
2015-2017[c]	Base	22.6 (20.3 to 24.9)	20.9 (19.4 to 22.5)	19.5 (17.6 to 21.4)	18.0 (16.2 to 19.7)	-1.7 (-4.4 to 1.1), P=0.24	-3.1 (-6.0 to -0.1), P=0.04	-4.6 (-7.5 to -1.8), P=0.002
	CDC guideline change[c]	21.5 (18.4 to 24.6)	20.1 (17.7 to 22.5)	17.3 (14.9 to 19.7)	15.7 (13.5 to 17.9)	-1.4 (-5.2 to 2.5), P=0.49	-4.2 (-8.1 to -0.3), P=0.04	-5.8 (-9.5 to -2.0), P=0.003
	Exclude the 1st Exam administration in 2008[b]	22.7 (20.4 to 25.0)	20.9 (19.4 to 22.5)	19.5 (17.6 to 21.5)	18.1 (16.3 to 19.8)	-1.8 (-4.5 to 0.9), P=0.20	-3.2 (-6.1 to -0.2), P=0.04	-4.7 (-7.5 to -1.8), P=0.002
	No training regression controls	23.1 (20.7 to 25.4)	21.3 (19.7 to 22.8)	19.7 (17.7 to 21.6)	18.0 (16.2 to 19.8)	-1.8 (-4.6 to 1.0), P=0.21	-3.4 (-6.4 to -0.3), P=0.03	-5.1 (-8.0 to -2.2), P=0.001
						Difference-in-differences: Quartile difference comparing 2015-2017[c] to 2012-2014[b]		
	Base					-0.8 (-4.4 to 2.9), P=0.68	-3.6 (-7.3 to 0.2), P=0.06	-4.1 (-7.8 to -0.3), P=0.03

© 2021 Gray BM et al. *JAMA Network Open*.

	CDC guideline change[c]		-0.4 (-5.0 to 4.1), P=0.85	-4.6 (-9.2 to 0.0), P=0.05	-5.1 (-9.6 to -0.6), P=0.03
	Exclude the 1st Exam administration in 2008[b]		-0.9 (-4.5 to 2.7), P=0.63	-3.7 (-7.5 to 0.1), P=0.06	-4.2 (-8.0 to -0.4), P=0.03
	No training regression controls		-0.8 (-4.5 to 2.8), P=0.66	-3.8 (-7.6 to 0.1), P=0.06	-4.3 (-8.1 to -0.5), P=0.03
			Difference-in-differences: Quartile difference comparing 2015-2017 [c] to 2009-2011		
	Base		-1.9 (-5.4 to 1.7), P=0.30	-3.9 (-7.7 to -0.1), P=0.04	-5.1 (-8.9 to -1.4), P=0.008
	CDC guideline change[c]		-1.6 (-6.1 to 2.9), P=0.49	-5.0 (-9.6 to -0.4), P=0.03	-6.3 (-10.8 to -1.8), P=0.006
	Exclude the 1st Exam administration in 2008[b]		-2.1 (-5.8 to 1.7), P=0.28	-4.5 (-8.6 to -0.5), P=0.03	-4.7 (-8.7 to -0.7), P=0.02
	No training regression controls		-2.1 (-5.7 to 1.5), P=0.25	-4.1 (-7.9 to -0.2), P=0.04	-5.3 (-9.1 to -1.4), P=0.007

[a] Regression adjusted means by quartile within year groups were constructed assuming a given quartile and year group interaction, holding all other covariates constant, and predicting prescriptions rates over the whole sample, [b]For the CDC sensitivity this time period is 2012 to 2016 <=April 16, [c]For the CDC sensitivity this time period 2016 >April 16 to 2017

© 2021 Gray BM et al. *JAMA Network Open*.

eTable 7. Regression Sensitivities for High Dosage or Long Duration

Years	Sensitivity Type	Regression adjusted mean percent by knowledge quartile, (95% CI)[a]				Percentage point difference compared to quartile 1, 95% CI, P-value		
		Quartile 1 (bottom)	Quartile 2	Quartile 3	Quartile 4 (top)	Quartile 2	Quartile 3	Quartile 4
2009-2011	Base	17.8 (16.1 to 19.5)	17.8 (16.6 to 19.0)	17.9 (16.5 to 19.3)	17.7 (16.2 to 19.2)	0.1 (-2.0 to 2.1), P=0.95	0.2 (-2.0 to 2.3), P=0.89	-0.1 (-2.4 to 2.2), P=0.94
	CDC guideline change	17.8 (16.1 to 19.5)	17.8 (16.6 to 19.0)	17.9 (16.5 to 19.3)	17.7 (16.2 to 19.2)	0.1 (-2.0 to 2.1), P=0.95	0.2 (-2.0 to 2.3), P=0.89	-0.1 (-2.4 to 2.2), P=0.94
	Exclude the 1st Exam administration in 2008	17.8 (15.8 to 19.8)	17.9 (16.6 to 19.2)	18.3 (16.8 to 19.9)	17.1 (15.6 to 18.7)	0.1 (-2.2 to 2.5), P=0.93	0.5 (-1.9 to 3.0), P=0.67	-0.7 (-3.2 to 1.8), P=0.59
	No training regression controls	17.8 (16.1 to 19.5)	17.9 (16.7 to 19.1)	17.7 (16.4 to 19.1)	17.3 (15.8 to 18.9)	0.1 (-1.9 to 2.2), P=0.90	0.0 (-2.2 to 2.2), P=0.99	-0.4 (-2.7 to 1.8), P=0.72
2012-2014[b]	Base	17.7 (16.0 to 19.4)	16.8 (15.5 to 18.1)	18.3 (16.9 to 19.7)	17.3 (15.9 to 18.8)	-0.9 (-3.1 to 1.2), P=0.39	0.6 (-1.6 to 2.8), P=0.61	-0.4 (-2.6 to 1.8), P=0.73
	CDC guideline change[b]	22.6 (20.5 to 24.8)	19.4 (18.1 to 20.7)	21.6 (20.2 to 23.0)	23.4 (21.7 to 25.1)	-1.1 (-3.1 to 0.9), P=0.27	-0.1 (-2.2 to 1.9), P=0.89	-1.1 (-3.2 to 1.0), P=0.32
	Exclude the 1st Exam administration in 2008	17.7 (16.0 to 19.4)	16.7 (15.4 to 18.1)	18.3 (16.9 to 19.7)	17.4 (15.9 to 18.8)	-1.0 (-3.1 to 1.2), P=0.38	0.6 (-1.7 to 2.8), P=0.62	-0.4 (-2.6 to 1.9), P=0.76
	No training regression controls	17.8 (16.0 to 19.5)	16.8 (15.5 to 18.1)	18.2 (16.8 to 19.6)	17.1 (15.6 to 18.5)	-1.0 (-3.2 to 1.2), P=0.37	0.4 (-1.8 to 2.7), P=0.72	-0.7 (-3.0 to 1.5), P=0.54
						Difference-in-differences: Quartile difference comparing 2012-2014 to 2009-2011		
	Base					-1.0 (-4.0 to 2.0), P=0.51	0.4 (-2.7 to 3.5), P=0.79	-0.3 (-3.5 to 2.9), P=0.85
	CDC guideline change[b]					-1.1 (-4.0 to 1.8), P=0.46	-1.2 (-4.3 to 1.9), P=0.44	-1.9 (-5.1 to 1.3), P=0.25
	Exclude the 1st Exam administration in 2008					-1.1 (-4.3 to 2.1), P=0.51	0.0 (-3.3 to 3.4), P=0.99	0.3 (-3.0 to 3.7), P=0.84
	No training regression controls					-1.1 (-4.1 to 1.9), P=0.46	0.4 (-2.7 to 3.6), P=0.79	-0.3 (-3.5 to 2.9), P=0.86
2015-2017[c]	Base	19.2 (17.1 to 21.4)	18.3 (16.8 to 19.8)	16.4 (14.7 to 18.2)	14.5 (12.9 to 16.1)	-0.9 (-3.5 to 1.6), P=0.47	-2.8 (-5.5 to -0.1), P=0.04	-4.8 (-7.4 to -2.1), P=<.001
	CDC guideline change[c]	21.5 (18.4 to 24.6)	20.1 (17.7 to 22.5)	17.3 (14.9 to 19.7)	15.7 (13.5 to 17.9)	-1.4 (-5.2 to 2.5), P=0.49	-4.2 (-8.1 to -0.3), P=0.04	-5.8 (-9.5 to -2.0), P=0.003
	Exclude the 1st Exam administration in 2008[b]	19.3 (17.2 to 21.4)	18.3 (16.8 to 19.8)	16.4 (14.6 to 18.2)	14.5 (12.9 to 16.1)	-1.0 (-3.5 to 1.6), P=0.45	-2.9 (-5.7 to -0.2), P=0.04	-4.8 (-7.4 to -2.2), P=<.001
	No training regression controls	19.8 (17.6 to 21.9)	18.7 (17.1 to 20.3)	16.5 (14.7 to 18.3)	14.4 (12.8 to 16.0)	-1.1 (-3.7 to 1.6), P=0.43	-3.2 (-6.0 to -0.4), P=0.02	-5.4 (-8.0 to -2.7), P=<.001
						Difference-in-differences: Quartile difference comparing 2015-2017 to 2012-2014		
	Base					0.1 (-3.3 to 3.4), P=0.98	-3.3 (-6.8 to 0.2), P=0.06	-4.3 (-7.7 to -0.9), P=0.01
	CDC guideline change[c]					0.9 (-3.3 to 5.1), P=0.67	-3.1 (-7.3 to 1.1), P=0.15	-4.5 (-8.6 to -0.4), P=0.03

© 2021 Gray BM et al. *JAMA Network Open*.

	Exclude the 1st Exam administration in 2008[b]		0.0 (-3.3 to 3.4), P=0.98	-3.4 (-6.9 to 0.1), P=0.06	-4.4 (-7.8 to -1.0), P=0.01
	No training regression controls		0.0 (-3.4 to 3.4), P=1.00	-3.6 (-7.2 to 0.0), P=0.05	-4.6 (-8.0 to -1.1), P=0.01
			Difference-in-differences: Quartile difference comparing 2015-2017 to 2009-2011		
	Base		-1.0 (-4.3 to 2.3), P=0.55	-3.0 (-6.5 to 0.6), P=0.10	-4.7 (-8.2 to -1.2), P=0.009
	CDC guideline change[c]		-1.6 (-6.1 to 2.9), P=0.49	-5.0 (-9.6 to -0.4), P=0.03	-6.3 (-10.8 to -1.8), P=0.006
	Exclude the 1st Exam administration in 2008[b]		-1.1 (-4.6 to 2.4), P=0.54	-3.5 (-7.2 to 0.3), P=0.07	-4.1 (-7.8 to -0.5), P=0.03
	No training regression controls		-1.2 (-4.6 to 2.2), P=0.49	-3.2 (-6.8 to 0.4), P=0.08	-5.0 (-8.5 to -1.5), P=0.006

[a] Regression adjusted means by quartile within year groups were constructed assuming a given quartile and year group interaction, holding all other covariates constant, and predicting prescriptions rates over the whole sample,

[b]For the CDC sensitivity this time period is 2012 to 2016 <=April 16,

[c]For the CDC sensitivity this time period 2016 >April 16 to 2017

eTable 8. Regression Sensitivities for Short Duration and Low Dosage

	Sensitivity Type	Regression adjusted mean percent by knowledge quartile, (95% CI)[a]				Differences compared with knowledge quartile 1 Percentage point difference, 95% CI, P-value		
		Quartile 1 (bottom)	Quartile 2	Quartile 3	Quartile 4 (top)	Quartile 2	Quartile 2	Quartile 4
2009-2011	Base	3.4 (3.4 to 4.8)	4.3 (3.7 to 4.9)	4.9 (4.2 to 5.6)	4.7 (4.0 to 5.4)	0.2 (-0.7 to 1.1), P=0.64	0.8 (-0.2 to 1.7), P=0.11	0.6 (-0.4 to 1.5), P=0.23
	CDC guideline change	4.1 (3.4 to 4.8)	4.3 (3.7 to 4.9)	4.9 (4.2 to 5.6)	4.7 (4.0 to 5.4)	0.2 (-0.7 to 1.1), P=0.64	0.8 (-0.2 to 1.7), P=0.11	0.6 (-0.4 to 1.5), P=0.23
	Exclude the 1st Exam administration in 2008	4.1 (3.4 to 4.8)	4.3 (3.6 to 5.0)	5.0 (4.2 to 5.7)	4.8 (4.0 to 5.5)	0.2 (-0.7 to 1.2), P=0.64	0.9 (-0.1 to 1.9), P=0.09	0.7 (-0.3 to 1.7), P=0.19
	No training regression controls	4.1 (3.4 to 4.7)	4.2 (3.6 to 4.9)	4.8 (4.1 to 5.5)	4.6 (3.9 to 5.3)	0.2 (-0.7 to 1.1), P=0.66	0.8 (-0.2 to 1.7), P=0.11	0.6 (-0.3 to 1.5), P=0.22
2012-2014[b]	Base	3.5 (3.5 to 5.3)	4.0 (3.5 to 4.5)	4.1 (3.5 to 4.8)	4.6 (3.9 to 5.3)	0.0 (-0.8 to 0.9), P=0.94	0.2 (-0.7 to 1.1), P=0.62	0.2 (-0.8 to 1.1), P=0.72
	CDC guideline change[b]	4.4 (3.5 to 5.3)	4.0 (3.5 to 4.5)	4.1 (3.5 to 4.8)	4.6 (3.9 to 5.3)	0.0 (-0.8 to 0.9), P=0.94	0.2 (-0.7 to 1.1), P=0.62	0.2 (-0.8 to 1.1), P=0.72
	Exclude the 1st Exam administration in 2008	4.2 (3.4 to 5.0)	4.4 (3.8 to 5.0)	4.3 (3.6 to 4.9)	4.2 (3.5 to 4.9)	0.2 (-0.8 to 1.2), P=0.72	0.1 (-1.0 to 1.1), P=0.91	0.0 (-1.1 to 1.1), P=0.98
	No training regression controls	4.2 (3.4 to 5.0)	4.3 (3.7 to 4.9)	4.2 (3.5 to 4.9)	4.2 (3.5 to 4.9)	0.1 (-0.9 to 1.1), P=0.79	0.0 (-1.0 to 1.1), P=0.95	0.0 (-1.1 to 1.1), P=0.99
						Quartile difference comparing 2012-2014 to 2009-2011		
	Base					-0.2 (-1.4 to 1.0), P=0.72	-0.7 (-1.9 to 0.6), P=0.30	-0.5 (-1.8 to 0.7), P=0.42
	CDC guideline change[b]					-0.2 (-1.4 to 1.0), P=0.72	-0.7 (-1.9 to 0.6), P=0.30	-0.5 (-1.8 to 0.7), P=0.42
	Exclude the 1st Exam administration in 2008					0.0 (-1.4 to 1.3), P=0.95	-0.8 (-2.3 to 0.6), P=0.27	-0.7 (-2.2 to 0.8), P=0.37
	No training regression controls					-0.1 (-1.4 to 1.3), P=0.93	-0.7 (-2.2 to 0.7), P=0.30	-0.6 (-2.0 to 0.8), P=0.42
2015-2017[c]	Base	3.0 (2.6 to 5.2)	2.5 (1.7 to 3.3)	2.4 (1.4 to 3.3)	2.9 (2.0 to 3.9)	-1.4 (-2.8 to 0.1), P=0.07	-1.5 (-3.0 to 0.1), P=0.06	-0.9 (-2.5 to 0.6), P=0.22
	CDC guideline change[c]	3.9 (2.6 to 5.2)	2.5 (1.7 to 3.3)	2.4 (1.4 to 3.3)	2.9 (2.0 to 3.9)	-1.4 (-2.8 to 0.1), P=0.07	-1.5 (-3.0 to 0.1), P=0.06	-0.9 (-2.5 to 0.6), P=0.22

© 2021 Gray BM et al. *JAMA Network Open*.

	Exclude the 1st Exam administration in 2008[b]	3.4 (2.5 to 4.3)	2.6 (2.0 to 3.1)	3.2 (2.5 to 3.9)	3.4 (2.7 to 4.2)	-0.8 (-1.9 to 0.2), P=0.11	-0.2 (-1.3 to 0.9), P=0.74	0.0 (-1.1 to 1.2), P=0.96
	No training regression controls	3.3 (2.4 to 4.2)	2.5 (2.0 to 3.1)	3.2 (2.5 to 3.9)	3.5 (2.7 to 4.2)	-0.8 (-1.8 to 0.2), P=0.13	-0.1 (-1.2 to 1.0), P=0.86	0.2 (-1.0 to 1.3), P=0.79
						Quartile difference comparing 2015-2017 to 2012-2014		
	Base					-0.9 (-2.3 to 0.5), P=0.20	-0.2 (-1.7 to 1.3), P=0.83	0.1 (-1.5 to 1.6), P=0.90
	CDC guideline change[c]					-1.5 (-3.2 to 0.2), P=0.09	-1.5 (-3.3 to 0.3), P=0.11	-0.9 (-2.7 to 1.0), P=0.35
	Exclude the 1st Exam administration in 2008[b]					-1.0 (-2.4 to 0.4), P=0.16	-0.3 (-1.8 to 1.3), P=0.74	0.0 (-1.6 to 1.6), P=0.99
	No training regression controls					-0.9 (-2.3 to 0.5), P=0.21	-0.1 (-1.6 to 1.4), P=0.86	0.1 (-1.4 to 1.7), P=0.86
						Quartile difference comparing 2015-2017 to 2009-2011		
	Base					-1.6 (-3.3 to 0.1), P=0.07	-2.3 (-4.0 to -0.5), P=0.01	-1.5 (-3.3 to 0.2), P=0.09
	CDC guideline change[c]					-1.6 (-3.3 to 0.1), P=0.07	-2.3 (-4.0 to -0.5), P=0.01	-1.5 (-3.3 to 0.2), P=0.09
	Exclude the 1st Exam administration in 2008[b]					-1.1 (-2.5 to 0.3), P=0.14	-1.1 (-2.6 to 0.4), P=0.16	-0.7 (-2.2 to 0.9), P=0.40
	No training regression controls					-1.0 (-2.3 to 0.4), P=0.16	-0.9 (-2.3 to 0.6), P=0.23	-0.4 (-1.9 to 1.0), P=0.56

[a] Regression adjusted means by quartile within year groups were constructed assuming a given quartile and year group interaction, holding all other covariates constant, and predicting prescriptions rates over the whole sample,

[b]For the CDC sensitivity this time period is 2012 to 2016 <=April 16,

[c]For the CDC sensitivity this time period 2016 >April 16 to 2017

© 2021 Gray BM et al. *JAMA Network Open*.

eReferences

1. Sun E, Moshfegh J, Rishel CA, Cook CE, Goode AP, George SZ. Association of early physical therapy with long-term opioid use among opioid-naive patients with musculoskeletal pain. *JAMA network open.* 2018;1(8):e185909-e185909.
2. Kolen MJ, Brennan RL. *Test equating, scaling, and linking: methods and practices, 2nd edition*. New York, NY: Springer-Verlag; 2004.
3. De Champlain AF. A primer on classical test theory and item response theory for assessments in medical education. *Med Educ.* 2010;44(1):109-117.
4. McDonagh MS, Selph SS, Buckley DI, et al. Nonopioid Pharmacologic Treatments for Chronic Pain. Comparative Effectiveness Review No. 228. (Prepared by the Pacific Northwest Evidence-based Practice Center under Contract No. 290-2015-00009-I.). *AHRQ Publication No. 20-EHC010. Rockville, MD* 2020; https://effectivehealthcare.ahrq.gov/products/nonopioid-chronic-pain/research. Accessed December 7, 2020.
5. Florida Health. Alternatives to Opioids:Medications. 2020; http://www.floridahealth.gov/programs-and-services/non-opioid-pain-management/_documents/alternatives-facts-8.5x11-eng.pdf. Accessed December7, 2020.
6. American Society of Anesthesiologists, Foundation A. Treatments to relieve chronic pain 2018; https://www.choosingwisely.org/wp-content/uploads/2018/02/Medicines-To-Relieve-Chronic-Pain-ASA.pdf. Accessed December 7, 2020.
7. By the American Geriatrics Society Beers Criteria Update Expert P. American Geriatrics Society 2019 Updated AGS Beers Criteria(R) for Potentially Inappropriate Medication Use in Older Adults. *J Am Geriatr Soc.* 2019;67(4):674-694.

© 2021 Gray BM et al. *JAMA Network Open*.
